# Provision and experience of care among women with hypertension in pregnancy: a multi-center qualitative study in Ghana

**DOI:** 10.1186/s12978-023-01593-0

**Published:** 2023-03-25

**Authors:** Kwame Adu-Bonsaffoh, Evelyn Tamma, Adanna Nwameme, Phyllis Dako-Gyeke, Emmanuel Srofenyoh, Evelyn K. Ansah, Diederick E. Grobbee, Arie Franx, Joyce L. Browne

**Affiliations:** 1 Julius Global Health, Julius Center for Health Sciences and Primary Care, University Medical Center Utrecht, Utrecht University, Utrecht, The Netherlands; 2 Department of Obstetrics and Gynaecology, University of Ghana Medical School, Korle-Bu, P.O. Box 77, Accra, Ghana; 3 Holy Care Specialist Hospital, Accra, Ghana; 4 Department of Social and Behavioural Sciences, School of Public Health, University of Ghana, Accra, Ghana; 5 Department of Obstetrics and Gynaecology, Greater Accra Regional Hospital (Ridge), Accra, Ghana; 6 Institute for Health Research, University of Health and Allied Sciences, Ho, Ghana; 7 Department of Obstetrics and Gynecology, University Medical Center Utrecht, Utrecht, The Netherlands

**Keywords:** Hypertension in pregnancy, Quality of care, Experience of care, Maternal hypertension

## Abstract

**Background:**

Hypertensive disorders of pregnancy (HDP) remain a leading global health problem with complex clinical presentations and potentially grim birth outcomes for both mother and fetus. Improvement in the quality of maternal care provision and positive women’s experiences are indispensable measures to reduce maternal and perinatal adverse outcomes.

**Objective:**

To explore the perspectives and lived experiences of healthcare provision among women with HDP and the associated challenges.

**Methods:**

A multi-center qualitative study using in-depth interviews (IDIs) and focus group discussions (FGDs) was conducted in five major referral hospitals in the Greater Accra Region of Ghana between June 2018 and March 2019. Women between 26 and 34 weeks’ gestation with confirmed HDP who received maternity care services were eligible to participate. Thematic content analysis was performed using the inductive analytic framework approach.

**Results:**

Fifty IDIs and three FGDs (with 22 participants) were conducted. Most women were between 20 and 30 years, Akans (ethnicity), married/cohabiting, self-employed and secondary school graduates. Women reported mixed (positive and negative) experiences of maternal care. Positive experiences reported include receiving optimal quality of care, satisfaction with care and good counselling and reassurance from the health professionals. Negative experiences of care comprised ineffective provider–client communication, inappropriate attitudes by the health professionals and disrespectful treatment including verbal and physical abuse. Major health system factors influencing women’s experiences of care included lack of logistics, substandard professionalism, inefficient national health insurance system and unexplained delays at health facilities. Patient-related factors that influenced provision of care enumerated were financial limitations, chronic psychosocial stress and inadequate awareness about HDP.

**Conclusion:**

Women with HDP reported both positive and negative experiences of care stemming from the healthcare system, health providers and individual factors. Given the importance of positive women’s experiences and respectful maternal care, dedicated multidisciplinary women-centered care is recommended to optimize the care for pregnant women with HDP.

## Plain language summary

High blood pressure (hypertension) in pregnancy can have severe complications for both mother and fetus including loss of life. The outcome of pregnancy for women who develop hypertension during pregnancy can be improved by ensuring optimal quality of care. In this study, we explored the opinions and experiences of women whose pregnancies were affected by hypertension concerning the care they received during their recent admission at different hospitals in Ghana and the challenges they faced. In four major referral hospitals in the Greater Accra Region of Ghana, we interviewed the women and had focus group discussions. Women who were pregnant for 26 weeks up to 34 weeks and had hypertension in pregnancy were invited for inclusion in the study.

We conducted in-depth interviews with fifty women and three focus group discussions with 22 women. Most women who participated in the study were between 20 and 30 years old, Akans (ethnicity), married/cohabiting, self-employed and secondary school graduates. The women reported both positive and negative experiences of care during their admission at the hospitals. Examples of positive experiences were receiving good quality of care, satisfaction with care, and adequate counselling from the health workers. Examples of negative experiences were poor communication between the providers and affected women, inappropriate attitudes by the healthcare providers, and disrespectful treatment such as verbal and physical abuse. The major factors in the health system that influenced women’s experiences of care were lack of logistics, substandard professionalism, inefficient national health insurance system and long delays at health facilities prior to receiving treatment. The individual women’s factors that affected the quality of care included financial constraints, psychosocial stress and inadequate knowledge about hypertension during pregnancy.

In conclusion, we determined that women with hypertension in pregnancy experience both positive and negative aspects of care and these may be due to challenges associated with the healthcare system, health providers and women themselves. There is the need to ensure optimal quality and respectful maternity care considering the nature of hypertension in pregnancy. These women require dedicated hospital staff with significant  experience to improve the quality of care provided to women with hypertension in pregnancy.

## Introduction

The global maternal mortality ratio is estimated at 199 per 100,000 live births, with a lifetime risk of maternal death of 1 in 190. The lifetime risk is substantially higher in sub-Sahara Africa (1 in 38) compared to high-income countries (1 in 5400) indicating significant healthcare inequities [1]. Hypertensive disorders of pregnancy (HDP) or maternal hypertension is among the major causes of maternal mortality, with complex clinical presentations and potentially devastating birth outcomes for both the woman and fetus [2]. HDP-related maternal morbidity and mortality disproportionately affect low- and middle-income countries (LMICs) [3, 4] with approximately 1900 maternal deaths in high-income countries, compared to 20,900 maternal deaths in Sub-Saharan Africa [3].

Prevention of avoidable maternal deaths through improvement in obstetric and newborn care has been a long-standing priority for the World Health Organization (WHO) and the global agenda of the Millennium Development Goals (2001–2015) and Sustainable Development Goals (SDGs, 2015–2030). Overall, provision of care (coverage) has improved during antenatal, intrapartum and postpartum periods, globally. However, lagging improvements to optimize the quality of maternal care resulted in reductions in maternal mortality that fall short of the global ambitions [5]. For instance, the maternal mortality ratio in Ghana remains high at 308 per 100,000 live births despite demonstrable improvements (740 in 1990). [1] About 98% of pregnant women in Ghana receive antenatal care from skilled birth attendants and the institutional deliveries rate has increased from 54% in 2007 to 79% in 2017 [6, 7]. The proportion of maternal deaths attributed to hypertension in pregnancy has doubled over the past decade in the country and it is the second largest cause after hemorrhage [6].

The WHO defines quality of care as the extent to which healthcare services provided to individuals and patient populations improve desired health outcomes [5]. High quality of care is multidimensional and incorporates safety, effectiveness, timeliness, efficiency, equitability and usefulness to people [5, 8]. Thus, improvement in the quality of care is critical in achieving the SDG 3’s target of reducing the global maternal mortality to less than 70 per 100,000 live births [9]. Importantly, during provision of care, the rights and dignity of the women should be respected to promote positive pregnancy and childbirth experience [10, 11]. Disrespectful care is increasingly being identified as endemic in most maternity care settings with a direct negative impact on the quality of care and can constitute a significant disincentive to future health-seeking behavior of women [11, 12].

Recently, a WHO multi-country study with Ghana inclusive reported that over 40% of women experienced significant mistreatment including physical, verbal, stigmatization or discrimination [13]. A key recommendation from this study hinges on further research into a comprehensive understanding of the drivers and structural dimensions of disrespectful maternity care including socio-economic inequalities. As such, women’s perspectives and their actual experiences of care at health facilities are vital to improving the existing healthcare system and the quality care for HDPs in the country. Therefore, the main objective of this study was to explore women's perspectives on provision and lived experiences of care and identify specific challenges among women treated for hypertension during pregnancy in five health facilities in Ghana.

## Methods

### Study design and setting

This multi-center qualitative study using both in-depth interviews (IDIs) and focus group discussions (FGDs) was conducted in five major health facilities in the Greater Accra Region (GAR) of Ghana. The study sites were Kore-Bu Teaching Hospital (KBTH), Greater Accra Regional Hospital, La General Hospital, Lekma Hospital and Tema General Hospital. The Greater Accra Metropolitan Area of Ghana has a population of about 4 million inhabitants with different ethnic backgrounds. The antenatal care coverage by skilled health provider is about 97.5% comprising mainly midwives and doctors. The region records the highest facility-based childbirth (91.9%) in the country with the majority (71.4%) from public institutions and about 20.5% from private health facilities [6].

This qualitative synthesis was part of a large study titled “Severe Preeclampsia adverse Outcome Triage study (SPOT study)". The overarching aim of the SPOT study was to validate the fullPIERS (Pre-eclampsia Integrated Estimate of RiSk) and miniPIERS risk prediction Models for adverse pregnancy outcomes in women with severe preeclampsia in Ghana [14, 15]. The detailed methodology of the SPOT study including the maternal outcomes has been published recently [16]. The main objective of the qualitative analyses was to comprehensively explore the quality of care for women with maternal hypertension in the clinical setting based on the lived experiences of pregnant women and perspectives of health workers. The health professionals’ perspectives on clinical challenges associated with managing maternal hypertension and context-specific recommendations have been published recently [17]. In addition, hypertensive mother’s knowledge, attitudes and misconceptions on HDP have been reported [18]. In this paper, we report hypertensive mothers’ perspectives and their lived experiences of care at health facilities in Ghana.

### Participants

Eligibility criteria were women with HDP diagnosed at gestational ages between 26 and 34 weeks, who received maternity services in any of the study centers and provided written informed consent. HDPs diagnosed before 34 weeks (early onset type) are considered severe disease with increased risk for poor outcomes and hospitalization for an extended period. We excluded women with hypertensive disorders diagnosed after gestational ages more than 34 weeks. There is evidence that planned early delivery for women with HDP after 34 weeks’ gestation is associated with less composite maternal morbidity and mortality compared with prolongation of the pregnancy [19]. Hypertensive pregnancies that occurred prior to 26 weeks were also excluded as conservative clinical management (i.e. prolongation of pregnancy) is generally not recommended due to the high risk of poor pregnancy outcomes [20].

### Participant recruitment and interviews

Data collection commenced on 1st June 2018 and was completed on 31st March 2019. Study participants were recruited via purposive sampling based on the specified inclusion criteria. Initially, a potential participants' list was compiled comprising women with hypertension in pregnancy. Patients that met the inclusion criteria were then identified by one of the authors (ET) with the help of the medical doctor in the study team. The selected potential participants were approached by ET who explained the study protocol to them individually. Women who agreed to participate in the study and provided informed consent were then assigned study identification numbers. The in-depth interviews (IDIs) were carried out immediately after discharge from the hospital. However, if IDI was missed after discharge from the hospital, the interview was re-scheduled within the postnatal period (six weeks postpartum). The IDIs were started first and continued until the point of saturation where no new information emerged from subsequent interviews. All the in-depth interviews (IDIs) were conducted by ET with regular supervision and support from KAB. The FGDs were also conducted and moderated by ET and notes were taken by another trained research assistant. We used interview guides for the IDIs and FGDs to gain a comprehensive understanding of the challenges during provision of care and experiences of hypertensive mothers. Both the IDIs and the FGDs were either conducted in Ga or Twi (local Ghanaian languages) and all were audio-recorded. The notes taken during the interviews were kept in a diary and provided additional clarification and greater transparency during the data analysis.

The IDIs and the FGDs were conducted in designated quiet rooms specifically allocated for the qualitative interviews in each facility to avoid frequent interruptions. There were no other people present in the interview rooms at the time of data collection apart from ET and the research assistant (note taker). The IDIs and FGDs usually lasted for between 30 to 60 min and 60 to 120 min respectively. The FGDs were conducted after the women had been discharged from the health facilities and within six weeks of childbirth so as to reduce recall bias and provide a clear picture of the overall quality of care they received during their admission at the health facilities.

### Ethical consideration

The study protocol was reviewed and approved by the Ghana Health Service Ethics Review Committee (Protocol ID GHSERC- GHSERC015 /09/17) and Ethical and Protocol Review Committee (EPRC) of the College of Health Sciences, University of Ghana (Protocol ID GHSERC- CHS-EtM.4-P1.2/2017-2018). We obtained written informed consent from all the study participants prior to the interviews and they were assured of strict confidentiality of the information provided. Anonymity was ensured by the non-inclusion of any identifiable information about the respondents.

### Data management and analysis

In this study, mixed methodological orientations of phenomenology and grounded theory were employed via systematic data collection and careful thematic content analysis [21]. We used an inductive analytic framework approach in the data analysis. In the inductive thematic analysis, the themes were derived mainly via coding of the data (data-driven) without being influenced by our theoretical interest in the topic. Deductive analytic approach complemented the analysis as data coding was not performed without any prior theoretical and epistemological background [21].

Transcription of the interviews and translation from Twi or Ga into English started soon after the commencement of the data collection and continued alongside the interviews. Prior to the data analysis, a two-day qualitative data analysis training session was organized for ET and KAB at the School of Public Health, University of Ghana, by the Social Scientists in the team headed by (PG and NA). After the training, the codebook was developed by ET with input from KAB based on the semi-structured interview guides. The transcripts were read multiple times by two authors (KAB and ET) in a more recursive manner to familiarize themselves with the data and to understand the train of thoughts of the respondents. During the recursive process of reading the transcripts, important notes were taken to indicate potential thematic areas and this resulted in the generation of the initial codes which were critical for the final coding of the transcripts. Coding was done by ET and KAB using NVivo software (version 12) based on the thematic content. During the data analysis, the notes that were scribed during the interviews provided clearly objective contribution and understanding via comparison with the transcripts. The study team discussed the codes and the emerging thematic areas until a consensus was reached.

In this study, triangulation of the results was ensured via the inclusion of hypertensive mothers of different backgrounds, from different health facilities (data source triangulation) and with the use of both IDIs and FGDs (method triangulation) [22]. Coding was undertaken by two authors (ET and KAB) and disagreements regarding coding were resolved via discussions by the team. The interviews were undertaken with a clear understanding of the principle of reflexibility and active note-taking during the IDI and FGDS. Reflexivity was ensured via comparison of the interview transcripts with the notes taken during the data collection to provide objective representation and greater transparency of the findings. The consolidated criteria for reporting qualitative research (COREQ) were used as a guide in reporting this paper [23].

## Results

### Characteristics of the study participants

**Fig. 1:**
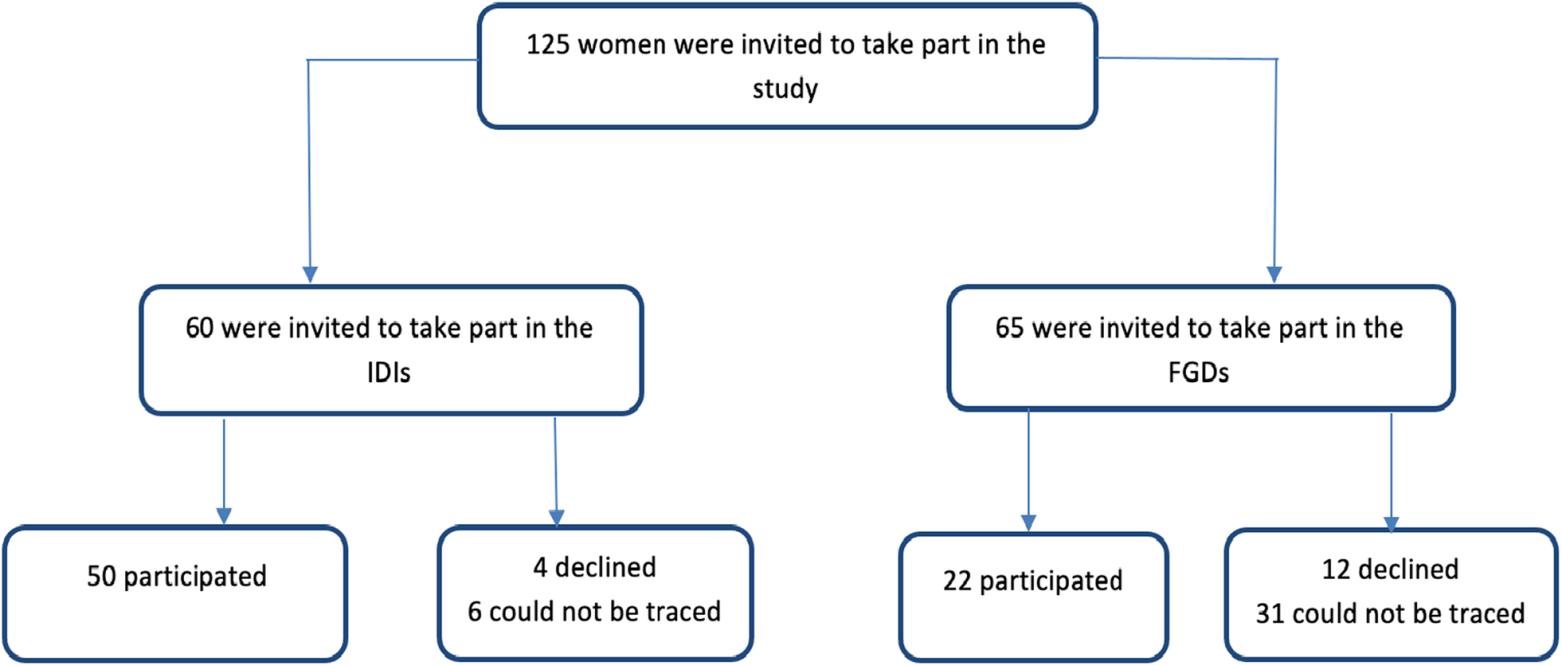
Flow chart for inclusion of women with hypertension in pregnancy

In this multicenter study comprising five hospitals in Ghana, a total of 125 women were invited to take part out of which 72 women finally participated comprising 50 and 22 for the IDIs and FGDs respectively. For the FGDs, most of the women could not be traced following discharge from the hospital (Fig. 1). The FGDs were conducted in three out of the five hospitals: Korle-Bu Teaching Hospital (n = 4 participants, 19 could not be traced out of 23 women invited), Greater Accra Regional Hospital (n = 10 participants, 5 could not be traced out of 15 women invited) and Tema General Hospital (n = 8 participants, 7 could not be traced out of 15 women invited). Overall, 12 women (18.5%) declined to participate in the FGDs. A total of 31 (47.7%) women (out of 65) could not be traced during the postpartum period following invitation to participate in the FGDs. There was some challenges in recruiting participants from the two smaller hospitals (La General hospital and Lekma hospital) for the FGD as we could not assemble the minimum number for the FGD on different occasions. The socio-demographic characteristics of the participants and the facility distributions are presented in Table 1.

**Table 1:** Socio-demographic characteristics of the study participants

Variable	IDIs n (%)	FGDs n (%)	Total n (%)
Age			
< 20	3 (6.0)	0	3 (4.2)
20–30	29 (58.0)	8 (36.4)	37 (51.4)
30–39	14 (28.0)	10 (45.5)	24 (33.3)
40 +	4 (8.0)	4 (18.2)	8 (11.1)
Marital status			
Single	17 (34.0)	5 (22.7)	22 (30.6)
Married/cohabiting	33 (66.0)	17 (77.3)	50 (69.4)
Educational status			
None/primary	14 (28.0)	3 (13.6)	17 (23.6)
Secondary	27 (54.0)	15 (68.2)	42 (58.3)
Tertiary	9 (18.0)	4 (18.2)	13 (4.2)
Number of previous births			
0	6 (12.0)	0 (0)	6 (8.3)
1–4	41 (82.0)	21 (95.5)	62 (86.1)
5 +	3 (6.0)	1 (4.5)	4 (5.6)
Residence			
Urban	48 (96.0)	22 (100.0)	70 (97.2)
Peri-urban	2 (4.0)	0 (0)	2 (2.8)
Ethnicity			
Akan	28 (56.0)	7 (31.8)	35 (48.6)
Ewe	10 (20.0)	6 (27.3)	16 (22.2)
Ga	6 (12.0)	4 (18.2)	10 (13.9)
Other	6 (12.0)	5 (22.7)	11 (15.3)
Occupation			
Unemployed	15 (30.0)	2 (9.1)	17 (23.6)
Formally employed	6 (12.0)	0 (0)	6 (8.3)
Self-employed	26 (52.0)	19 (86.4)	45 (62.5)
Casual worker	1 (2.0)	0 (0)	1 (1.4)
Others	2 (4.0)	1 (4.5)	3 (4.2)
Health facilities (study sites)			
Korle-Bu Teaching Hospital	20 (40.0)	4 (18.2)	24 (33.3)
La-General Hospital	4 (8.0)	–	4 (5.6)
Lekma Hospital	5 (10.0)	–	5 (6.9)
Greater Accra Regional Hospital	16 (32.0)	10 (45.5)	36 (50.0)
Tema General Hospital	5 (10.0)	8 (36.4)	13 (18.1)

*IDIs* in-depth interviews, *FGDs* focus group discussions

Most of the women included in this study had Akan ethnicity (48.6%, n = 35), and were married/co-habiting (69.4%, n = 50), self-employed (62.5%, n = 45) and between the age group of 20 to 30 years (51.4%, n = 37). Majority had attained secondary education (58.3%, n = 42) and experienced between 1 to 4 previous childbirths (86.1%, n = 62) and lived in urban areas in the Accra Metropolis (97.2%, n = 70). Majority of the IDIs were contributed by the Korle-Bu Teaching Hospital (40.0%, n = 20) and Greater Accra Regional Hospital (32.0%, n = 16). Of the 60 potential participants for the IDIs, ten (16.7%) were excluded (4 declined and 6 could not be traced).

In this study, we explored the women’s perspective on provision and experiences of care, and specific challenges faced by women treated for maternal hypertension. The major themes that emerged included (1) women’s knowledge on hypertension in pregnancy, (2) women’s experiences of care and (3) challenges experienced by women while receiving care.

#### 1. Women’s knowledge on hypertension in pregnancy

Most of the study participants had limited knowledge about HDP including the danger symptoms of severe hypertension, especially those with limited educational level. Most women indicated that they were ignorant about preeclampsia and other HDPs and wondered why health workers do not routinely educate them on the subject.

*“Please, I will like to ask that the name that they are mentioning [pre-eclampsia], is it an illness? (Laughter by the women) Because that is what the doctors always write. Because we are illiterates we don’t understand. I don’t know what it is” (FGD, 40 years, married)*

*“Truth be told, I had never heard about it before. And I still really don’t even know what it is in detail. I quite remember I even use to point to the wrong place when I was asked to point to my heart. In my first pregnancy, nothing about hypertension was mentioned to me (IDI, 29 years, single)*

However, few mothers had adequate knowledge of hypertension and its major complications including stroke. Adequate knowledge was commonly demonstrated among women who had experienced preeclampsia or other types of maternal hypertension in their previous pregnancies. Most women diagnosed with hypertension in pregnancy had limited knowledge about the condition before their diagnoses were made. Other women did not know that hypertension can affect pregnant women although they had heard about hypertension in the general population.

*“Your health is the most important because your BP [blood pressure], when it goes up very high it can kill you or leave you with a stroke” (IDI, 29 years, married)*

*“I’ve heard it because I experienced it in my previous pregnancy and I know what it can bring about. So when even someone says [s]he has headache I tell the person to go and check, it might be hypertension because it can kill you easily” (IDI, 39 years, single).*

*“I have heard about BP before but I didn’t know that you could have BP when pregnant” (IDI, 31 years, married)*

Majority of the respondents attributed their hypertension to stressful situations they experienced during pregnancy. Other women related the occurrence of the hypertension to grudges at workplaces and the home environment. Some participants hinted that in some situations, disturbances in the home environment by family members resulting in ‘excessive thinking’ in pregnancy were associated with hypertension in pregnancy. Most women frequently attributed hypertension in pregnancy to stressful situations which lead to heightened psychological stress and excessive thinking by the women.

*“Where I was staying, there were other tenants living there who always want to argue with me. Anytime they see me they start to insult and mock at me. So because of that I decided to leave that house because I was very angry so I don’t know if that is what triggered the BP” (IDI, 28 years, single).*

*“What I can say about it is that when we think too much that is what causes it so if you are thinking reduce it and give everything to God. He does all things but when you think too much it will not be able to solve that problem, and then also you should find time to rest. You shouldn’t do too much work” (IDI, 39 years, married).*

#### 2. Women’s experiences of care

Experience of care was a key recurring theme reported by majority of the women with maternal hypertension. The respondents had different interpretations of what was considered “good quality of care” based on their lived experiences of care at their respective health facilities and the outcomes of their pregnancies. The reported experiences by the women relating to their care at the respective health facilities were mixed. Few women reported positive experiences and perceptions of good quality of care. The hypertensive mothers narrated mixed feelings regarding their care experiences, indicating significant dissatisfaction among participants. However, some hypertensive mothers had positive experiences and described the quality of care they received as optimal. The high quality of care experienced by some of the women commenced with excellent reception at the health facilities followed by provision of appropriate treatment (standards of professionalism) and reassurance by the health workers.

*“As for me I was well cared for. They've really cared for me. The way the thing [hypertension] happened to me and the way they were able to take care of me. They gave me injections when they had to. They really took very good care of me and I’m very happy” (FGD, 28 years, married)*

*“On the day that I came, honestly, they gave me a good reception because my baby’s heartbeat was up and my Bp was also up so they calmed me down so that my Bp will come down. So they gave me excellent care and I was very happy” (FGD, 42 years, married)*

However, it was apparent that some women were extremely unhappy with the care they received while they were on admission. They enumerated the negative experiences they encountered and recommended measures to mitigate against such inappropriate treatment by health workers.

*“Getting up from the bed was very difficult for me. I could not raise my leg. A nurse will ask you to come to her for her to check your temperature and something else while she is seated at the other end. I couldn’t walk and almost fell so I had to hold unto the beds of others when I walked a little bit. On the other hand, there are those (nurses) who will come over to help you when you tell them you can’t get up” (FGD, 41 years, married).*

*“When labour started, at the initial stages when I called any nurse who was passing by, they ignored me instead of them may be encouraging me to bear the pain. When it happens like that you think that maybe you are going to die not knowing anybody there because the person who you know as a nurse who is supposed to help you isn’t. When the baby’s head was coming out she then asked me to get up. It’s fine if you don’t know the condition in which a woman has to go through when in labour. When I had squatted when the baby’s head was coming, she was looking on but she kept urging me to get up and I told her that I couldn’t get up in that condition. There was a container under my bed, and it was in this container that I delivered into (Respondents: ooh!). I was very hurt and told my husband. I was very hurt because I’m sure she was one of the student nurses. If she had drawn closer and helped me with the delivery maybe I wouldn’t have lost so much blood. I bled a lot and suffered a lot before the baby came out” (FGD, 26 years Married).*

There were mixed findings regarding provision of relevant information and counselling with respect to procedures undertaken by health professionals. Some women were given comprehensive counselling prior to the procedures they went through; they were satisfied with the care received and they commended the health professionals.

*“Yes, they will tell you this is going to be painful. They tell you before they inject you. When I came back from the theatre, I told the doctor that my buttocks really hurt. He told me that he will mix the drug with another drug to make it less painful. So he mixed it with another drug before injecting me and the pain was less” (IDI, 31 years, married).*

### a. Experience of complications of maternal hypertension

Although the pregnancy outcomes were generally good for most of the women, some experienced adverse outcomes of maternal hypertension, such as the demise of their babies. The narratives provided by some mothers who experienced adverse outcomes clearly indicated that they had some form of postpartum psychological strain and depression.

*“For me, since they took the child out I didn't want anyone to come to me because in my room when the babies around me cry, I panic, so I told them to let me go home” (IDI, 29 years, single)*

*“They did a scan and realized that the baby had died in my womb. My sister signed as a witness to the death of the baby. After my vitals and blood were checked, everything was alright except my BP” (IDI, 28 years, married)*

Some of the patients experienced severe complications of maternal hypertension such as convulsions or loss of consciousness (eclampsia). A typical example was a woman who collapsed (had eclampsia) and was rushed to the hospital in an unconscious state and was referred to the tertiary center for further treatment.

*“After collapsing at home, I was sent to a nearby clinic and after regaining consciousness the clinic transferred me here. When I got here, I was given medicine” (FGD, 26 years, married)*

A similar occurrence of eclampsia and prolonged loss of consciousness was reported by a young woman who regained her consciousness long after she had been operated upon (cesarean section).

*“I remember I started eating kenkey and started vomiting and that was it. I didn’t remember anything again…. I saw that there was plaster on my stomach and I was lying down before my mother came and I ask them what I was doing here and they said I had been operated because I was pregnant” (IDI, 18 years, single).*

### b. Experience of mistreatment and disrespectful care

Some of the participants recounted unpleasant experiences of disrespectful treatment while receiving maternal care services at the health institutions. These abusive treatments were meted by different categories of health professionals (doctors, midwives) and took different forms including verbal, neglect and physical mistreatment. Verbal abuse was rampant, and most women reported their experience of being shouted at, insulted or scolded during the provision of care. In addition, non-verbal abusive attitudes were displayed toward some hypertensive women.

*“Some people [health workers] talk to you in a “funny way” so you won't feel it but others too will be shouting at you and you think she is doing her job so you can't say anything about it, but some do it in a very nice way” (IDI, 28 years, single)*

*“In all the doctors take good care of us, but the nurses who work with the doctors are snobs. So you will say that I don’t like this place because when you go there the nurses are snobs. They do this too much” (IDI, 31 years, married)*

Various instances of neglect by healthcare providers were mentioned by the respondents. The affected women felt neglected and worried especially during the times when they needed the support of the health professionals most. Incidents of extreme forms of inadequate attention or abandonment by health workers during the critical times of childbirth in the health facilities were recounted with a lot of emotions by some of the women.

*“When I was in labour, I had to tell the nurse that she should come and check me so I can go to the labour ward and she only told me that I should allow them to sleep because that time it was around dawn, 2am. She said I should allow them to sleep and that I’m not in any labour. So I had to go and so it was when I started pushing that the people [other patients] on the ward called out to the nurses “she is giving birth ooo” and when they came I had given birth” (IDI, 24 years, married)*

In the process of provision of maternal care some women experienced physical abuse which included being hit by health providers. Reasons cited for such mistreatment include patients’ refusal to obey instructions and lack of patience on the health workers’ part.

*“When they [health workers] have to wake you up for you to take your medication, they hit you very hard as if you were their little sister before they will tell you to take your medication instead of tapping you gently. That was my problem” (FGD, 37 years, married)*

Sometimes the attitudes of some of the health workers put the patients off and made them wish they had an alternative health facility to seek treatment. Majority of the mothers felt uncomfortable and worried when health professionals who are taking care of them are unfriendly and disinterested in their welfare.

*“Some nurses are not all that friendly; if you are talking to them as if they are listening, sometimes the way they talk to you makes you feel uncomfortable, so I think at least they should be a little friendly to us” (IDI, 24 years, married)*

Lack of interactive communication between the health professionals and hypertensive women was considered a major shortcoming in the process of providing care. Adequate communication from the doctors and nurses on the status and progress of their medical conditions with heartfelt expression of empathy was a major expectation of the women. Some women observed that the lack of communication was even worse for women with no or minimal educational attainment.

*“Hmmm I have a problem with the doctors and nurses, some don’t explain things to the patients. You come and they say everything is okay and fine, unless those who have gone to school a little bit and can read. But I think in everything, they should tell the patients” (IDI, 26 years, married).*

### 3. Challenges experienced while receiving care

Women with hypertension in pregnancy experienced myriads of challenges related to the health system and attitudes of healthcare professionals while receiving care at health facilities. Institutional challenges include inadequate facilities such as beds or space for managing women referred for urgent care due to severe maternal hypertension. Unavailability of hospital beds for admitting mothers with hypertensive emergencies in most health facilities was frequently mentioned. However, urgent institutional arrangements were made in some cases to provide space for admission of the affected mothers following some avoidable institutional delays.

*“When we came in the evening, we were told there was no bed. So they came to look for a place to put a bed. So they cleared the place where they had put certain things and then put me there. They said because of my case I had to lie down. I shouldn’t be standing so they made a bed for me to lie down and then they checked my BP frequently” (IDI, 28 years, married)*

Situations where pregnant women on admission had no beds and slept on the floor were also mentioned. Such situations occurred frequently when the hospitals were overwhelmed with high patient loads. A typical example of these experiences encountered personally by some of the hypertensive mothers is indicated below.

*“Please one good experience I encountered when I was transferred after delivery to the ward was that, there were no beds for the first half and I was given a mattress which I laid on till evening. In the evening one midwife came and asked why a BP patient was lying on the floor. There was a bed then, so she carried me unto the bed like a baby. The woman [midwife] did very well. So, this is one good experience which I had” (FGD, 32 years, co-habiting)*

In some instances of “no bed syndrome”, some hypertensive pregnant women are managed in chairs until beds become available. The following quote describes a typical experience by one hypertensive pregnant woman who was nursed in a plastic chair when she presented with severe hypertension and required hospital admission and immediate treatment

*“When I arrived here, I was told there was no bed so I should look for a plastic seat and sit on. So I sat in the seat while they took care of me. I was injected and all that sitting in the chair” (FGD, 31 years, married)*

### a. High cost of laboratory tests and antihypertensive medications

The cost of healthcare was a prominent theme that emerged from almost all the respondents. It became clear that the most important underlying challenge associated with the care for women with maternal hypertension was financial constraint. An important concern mentioned by majority of the women was the high cost of hospital stay. In addition, the cost of medications (antihypertensive drugs) were high for which they implored the government and other organizations to support.

*“The drugs are very expensive. There are some drugs which are not covered by health insurance. You will have to buy it yourself. You can buy drugs to the tune of 400, 500, 600 and sometimes 1.2 cedis [with the sum mentioned here ranging from 70 to 125 USD]. Some are even more than that. So you will buy it yourself. Health insurance doesn’t cover. It covers very little, the ones that are not expensive like 35 or 5 cedis” (IDI, 33 years, married)*

Financial constraint was cited as the single most important challenge encountered by women with hypertension, especially in paying for their laboratory tests and medications. The participants made recommendations to the government to either reduce the cost of the medications or supply the relevant drugs to them at no cost.

*“So the government should make sure that the labs [laboratory tests] done for pregnant women with hypertensive disorders of pregnancy should be made free because without the labs the doctors cannot do their work well” (IDI, 33 years, married)*

*“The government should reduce the cost and help those of us who don’t have money so that we can receive the care the doctors are ready to give us. Because if you have high blood pressure and you don’t have money, you are still thinking how will the hypertension will go?” (IDI, 43 years, married).*

### b. Insecurity about the proficiency of the medical team

Some women with maternal hypertension had the impression that some of the medical practitioners were not adequately competent to offer optimal treatment to them on certain occasions. There were instances of arguments among the doctors about the most appropriate clinical decision in the presence of the patients and this created a feeling of insecurity, uncertainty and fear due to perceived impression of inexperienced medical personnel. These feelings of insecurity were compounded by lack of communication and interaction with the affected patients who only looked up to God for miracle. Some mothers narrated how they were scared by the actions or clinical decision of some doctors.

*“My problem over here is that it was like trial and error. When this person comes [referring to the doctor] he will come and write his report, “severe pre-eclampsia”. When this person [referring to another doctor] also comes, he writes mild pre-eclampsia and then leaves. What I have being yearning for my whole life [referring to a baby], you have students coming in and out. When this one comes, he comes to write then when the doctors come, they don’t read the report. This one comes to write “mild” and then the other one comes to write “severe”. So when the time came for me to go to theatre they should have found out whether it [the baby] will come on or not, but they were arguing among themselves that I was para zero or para “o” or something so they had to go and take the baby out for me. So this is the problem I had” (FGD, 42 years, married).*

*“When I came they [doctors] did not explain things to me and when they checked they asked me to go home and come the following week but if it had not been for the head of the hospital I could have gone home and something could have happened to me” (FGD, 29 years, single)*

### c. Delays in receiving care at health facilities

Majority of the mothers with maternal hypertension recounted their experiences of significant delays at the health facilities before receiving the needed care. This was reported by participants from all the health facilities included in the study. Further enquiry indicates that the actual provision of care they received at the facilities are commendable despite prior delays in accessing the care.

*“I leave home very early because I’m coming from afar. I get here by 6 am and start heading back home at 5 pm. We suffer a lot. We are cared for alright but some people who arrive later go ahead of us because they know someone who works here. We sit in the queue for long because we don’t know anyone who works here. So we really suffer a lot” (FGD, 31 years, married).*

Some women who were referred on account of severe hypertension had to obtain folders before they were provided the needed care. The challenge of going through the long registration process without the initial triage or treatment results in significant delay in receiving the needed urgent care, especially for emergency cases. Such long processes of acquiring folders (in-hospital registration) could result in increased adverse pregnancy outcomes for undiagnosed hypertensive emergency due to delay in initiating anti-hypertensive treatment on time.

*“When I got here, I was asked to go and make a card. After that I was given a nurse who checked my BP so upon checking my BP, they realized it was an emergency so they saw that the BP had gone up so they gave me to a doctor” (IDI, 33 years, married)*

*“We got here at 3 something and they asked us to go and get a folder. My husband kept long with the getting of the folder. So I was attended to after the folder was brought and they checked my Bp and then they sent me to a room. I was given injections instantly” (FGD, 34 years, Married)*

### d. Shortage of health professionals

Majority of the women thought the number of health professionals (nurses and doctors) were inadequate to take care of the high patient load. Most women appreciated implicitly that the actual number of health professionals were not adequate. However, they (health workers) were mostly supported by students who were learning on the job. Some patients attributed poor professional output by the health professionals to the high patient load and the inadequate numbers to manage the high volumes of obstetric cases.

*“I think sometimes there is pressure on them (doctors) and some of the patients they do not abide by the instructions, and they don’t take their drugs and come with worse conditions. So, I think a lot of doctors should be employed” (IDI, 26 years, married).*

*“The midwives are not many so if more of those who have completed school can be added. At the government hospitals the patients are more than the nurses that is why they don’t have time for us so the government should see to it for us” (FGD, 41 years, married)*

### e. National health insurance associated challenges

There was a general perception that the National Health Insurance (NHIS) has major limitations and does not cover the cost of maternal care completely. Most women had extreme difficulty in procuring all the prescribed medications and laboratory tests. These sentiments were expressed by almost all the hypertensive mothers with mixed reports on whether the NHIS covers the drugs and laboratory tests fully or partly.

*“The NHIS doesn’t cover the BP drugs so they should improve the insurance so that it covers the drugs because not everyone can afford it. When I came, I have spent almost 600 Ghana cedis here and not everyone can afford it” (IDI, 29 years, single)*

*“With the labs it was not expensive because the health insurance covers some and the rest you will have to pay for it. And the drugs too, the insurance covers some of them so the ones it does not cover you have to buy them either at the hospital when they have some or outside”. (IDI, 31 years, single)*

Based on the gross limitations associated with the NHIS as enumerated by the women including its inability to pay for the cost of most medications and laboratory tests, several requests were made to the government. These appeals mainly related to increasing the coverage of the NHIS to defray the hospital bill for women with maternal hypertension.

*“They should increase the coverage of the insurance. We are told with insurance, delivery is free but this is not so we still pay. Even for the labs and drugs we still pay. So far, I have spent almost 500 cedis” (IDI, 33 years, married)*

### f. Home care management

Some participants recommended the idea of managing some selected patients at home for less severe conditions as compared to the strict hospital confinement. Inferably, some women felt their condition were not severe enough to require admission to the hospital and that the possibility of home care and monitoring for less severe cases of maternal hypertension should be explored by health professionals. The reasons for advocating home management as compared to hospital care majorly related to reduced healthcare cost and loss of women’s productive time.

*“I think when someone comes and the condition is not too serious the doctors should prescribe drugs for the person and not necessarily admit the person as it takes some productive hours of work away from the person and being admitted here, the cost of admission too is expensive and not all can pay” (IDI, 29 years, single)*

*“For me, I was not ok because I told them to allow me to go home so that I can come for review while searching for some money and they refused, and it was because of my siblings I left at home” (IDI, 25 years, single)*

## Discussion

This multi-center qualitative study provides a unique opportunity to understand the quality of maternal care from the perspectives of women treated for hypertension during pregnancy and their lived experiences of care at health facilities in Ghana. Women with HDP reported mixed (positive and negative) experiences of care. Major bottlenecks in the provision of high-quality care identified relate to health system challenges such as lack of logistics, inefficient national health insurance and unexplained delays at health facilities; health professionals-related factors including ineffective provider–client communication, inappropriate attitude by the health professionals, disrespectful treatment including verbal and physical abuse; and inadequate women’s knowledge about hypertension in pregnancy.

The finding of inadequate knowledge on the preeclampsia or maternal hypertension by the hypertensive mothers is consistent with other reports across the globe especially in LMICs [24–28]. In a related study in the United Kingdom, Wotherspoon et al. determined limited knowledge of preeclampsia by women most of whom were unaware of the potential risk of developing preeclampsia [29]. In that study, majority of the women were uninformed about the rationale for regular measurement of their blood pressures and urine samples. Relatedly, majority of the mothers attributed the development of hypertension to stressful situations they experience during pregnancy especially from their workplaces and home environment. The notion of ‘stress-induced preeclampsia’ was of paramount concern to the hypertensive mothers and this emerging discovery requires further research as similar findings have been reported [28]. Although the etiology of maternal hypertensive remains elusive recent studies have demonstrated causal associations with chronic psychosocial stress [30–33].

Majority of the mothers with maternal hypertension recounted their experiences of significant delays at the health facilities before receiving healthcare services as reported by other studies [2, 34, 35]. These unnecessary and avoidable delays could result in preventable maternal deaths or severe morbidity [35, 36]. Also, high costs of laboratory tests and antihypertensive medications were considered a major challenge for women and continuous support from the government was frequently solicited. In this case, all-inclusive and effectively working NHIS is indispensable in improving the quality of care. Majority of the mothers lamented desperately on the limitations of the existing NHIS. Originally, the NHIS in Ghana was deemed to cover 95% of all healthcare cost (including maternal care services) with some specific exceptions [37]. However, the constant public outcry including reports of frustrations experienced by hypertensive mothers in this multi-center study calls for a critical review of patients’ benefit and coverage of the NHIS in the country.

Another important health system-related concern was the feeling of insecurity by some women about the proficiency of the medical teams. Some hypertensive mothers had the impression that some of the health workers were not adequately competent. Unavailability of skilled personnel to make correct diagnosis and implement appropriate healthcare plan constitutes substandard care with increased risk of severe maternal near-miss and mortality [38]. The challenge of substandard care for maternal hypertension is not limited to LMICs. For instance, in the Netherlands where maternal mortality ratio is among the lowest worldwide, maternal hypertension is the leading cause of maternal deaths with about 96% associated with substandard care [39]. Similar concerns related to the quality of maternal care have been reported in other high-income countries including the confidential inquiries into maternal deaths in the United Kingdom. The issue of substandard care remains a major clinical challenge that warrants urgent attention globally [27].

In addition, a significant number of mothers reported personal experiences of disrespectful treatments from some health professionals including neglect, verbal and physical abuse. Such mistreatment of women during provision of maternal care remains a global phenomenon with worse implications in the LMICS [11, 40]. In Ghana, disrespectful maternity care occurring in various forms has been reported with differing opinions about its rationale or acceptability in contemporary maternal care [12, 41–43]. Admittedly, mistreatment of women during provision of maternal care is a complex phenomenon that requires the input of all stakeholders including the government, health institutions, health professionals and society. More recently, a WHO multi-country study on mistreatment of women comprising both labour observation and postpartum community survey reported that over 40% of women experienced some form of mistreatment [13]. The main public health concern about mistreatment of women with hypertensive disorder relates to its potential to disincentivize prospective mothers and their families in seeking care at health facilities. Evidence-based interventions of locally appropriate dimensions are urgently required to minimize abusive treatment of women and improve respectful maternity care in the country.

Effective communication between health care providers and women is strongly recommended by WHO to enhance positive experience of care and minimize unnecessary anxiety [10]. In our study, inadequate interactive communication was a major theme that emerged from majority the hypertensive mothers, consistent with similar reports from other studies [25, 44, 45]. In a related study in the United States, various recommendations were provided to improve effective communication between patients and healthcare providers including building trust, rapport and reflective listening [46]. Lack of effective communication negatively impact on satisfaction with care. In a study conducted in Germany, approximately 70% of the hypertensive mothers reported dissatisfaction with the medical information provided by their healthcare providers on maternal hypertension [47]. This quantitative report by Leeners et al. [47] complements the qualitative reports demonstrated in our study and calls for a paradigm shift in provider–client communication. Re-training and empowerment of healthcare professionals, including improvement of their salaries, health facilities and personal circumstances are viable measures to improve efficient provision of care and women’s experiences.

Intriguingly, the concept of home care management and monitoring for less severe maternal hypertension was raised by some of the hypertensive mothers to reduce the burden on health facilities, health care cost and improve women’s productivity. In another study, Barlow et al. reported expression of similar recommendation by hypertensive mothers as they preferred to continue their bed rest and medications at home because they thought their condition were not severe [48]. In some high-income countries home management of women with non-severe maternal hypertension is permissible and recommended [49–51]. More recently, Perry et al. reported significant reduction in the number of hospital visits among hypertensive pregnant women when managed on home-based blood pressure monitoring without increasing adverse pregnancy outcomes [52].

### Strengths and limitations

The main strengths of this qualitative study include the multi-center nature comprising five major hospitals in Ghana and the rigorous methodology adopted. In this study, women of various age groups were sampled purposively which resulted in a comprehensive assessment of experiences of care and major determinants of quality of care. Another important aspect of this study relates to the timing of the interviews which occurred after hospital discharge which enabled participants to express the opinions freely without any fear of retribution from the healthcare providers. Also, all the IDIs and the FGDs were undertaken by a trained researcher who is non-healthcare professional, and this enhanced the women’s willingness to discuss their views freely. Finally, we employed both IDIs and FGDs to ensure comprehensive understanding (method triangulation) [22] of the process involved in the provision of care and lived experiences of hypertensive mothers.

This study has some limitations. Although this was a multi-center study, it was mainly conducted in the southern zone of the country where coverage of maternal healthcare services is highest. Therefore, women’s experiences of care including challenges associated with provision of maternal care services reported in our study may be underestimated. Also, data collection for the IDIs was mainly undertaken by only one author and this may have influenced the triangulation of the findings (investigator triangulation) with increased potential for monotony. Investigator triangulation relates to the use of more researchers in data collection or analysis resulting in improved assurance of data variety and confirmation of the findings [22]. However, the findings of this study depict the gross overview of the quality of care associated with maternal hypertension in the country.

## Conclusion

This multi-center qualitative study has highlighted hypertensive women’s perspectives on the quality of care and their lived experiences with the care for women with hypertensive disorders of pregnancy. A complex array of elements affects the provision and experience of care for women with maternal hypertension. This includes health system related factors such as *lack of logistics, substandard professional attitude and unexplained delays at health facilities*. Patient related factors that negatively influence the provision of care comprise inadequate awareness of maternal hypertension and its complications and financial challenges. The quality of care experienced by women with maternal hypertension was negatively influenced by ineffective provider–client communication, inappropriate attitude by the health professionals, disrespectful treatment.

The quality of provision and experience of care for maternal hypertension in the country could be improved by integration of appropriate evidence-based interventions at different levels such as health system, healthcare cost coverage, regular refresher courses for health workers and patient-centered care interventions. The emphasis should be placed on multidimensional collaboration of all stakeholders in both governmental and non-governmental organizations as well as the entire society. Well-integrated maternal health education promotion should be integrated into the educational programs to create and maintain optimal awareness about the relevance of high-quality maternal health. Further studies of high methodological quality with wider national coverage are recommended to better understand how quality and experience of care can be improved for women with maternal hypertension.

## Data Availability

The full transcripts for this qualitative study are available upon request from the corresponding author.

## Abbreviations

COREQ: Consolidated criteria for reporting qualitative research
FGD: Focus group discussion
GAR: Greater Accra Region
HDP: Hypertensive disorders in pregnancy
IDI: In-Depth interview
KBTH: Korle-Bu Teaching Hospital
LMICs: Low- and middle-income countries
NHIS: National Health Insurance Scheme
SDG: Sustainable Development Goals
WHO: World Health Organization

## References

[1] WHO, UNICEF, UNFPA WBG and the UNPD. Trends in maternal mortality 2000 to 2017. Geneva, 2019.

[2] Osungbade KO, Ige OK (2011). Public health perspectives of preeclampsia in developing countries: implication for health system strengthening. J Pregnancy.

[3] Say L, Chou D, Gemmill A (2014). Global causes of maternal death: a WHO systematic analysis. Lancet Glob Health.

[4] Duley L (2009). The global impact of pre-eclampsia and eclampsia. Semin Perinatol.

[5] World Health Organization (WHO). Standards for Improving Quality of Maternal and Newborn Care in Health Facilities. Geneva, www.who.int/iris/handle/10665/249155 (2016).

[6] Ghana Statistical Service (GSS), Ghana Health Service (GHS), ICF. Ghana Maternal Health Survey 2017. Accra, Ghana, 2018.

[7] Ghana Statistical Service (GSS), Ghana Health Service (GHS), Macro International.Ghana Maternal Health Survey 2007. Calverton, Maryland, USA, 2009.

[8] Lachman P, Batalden P, Vanhaecht K (2021). A multidimensional quality model: an opportunity for patients, their kin, healthcare providers and professionals to coproduce health. F1000Res.

[9] UN General Assembly. Report of the Open Working Group of the General Assembly on Sustainable Development Goals. 2014.

[10] World Health Organization (WHO). WHO recommendations: intrapartum care for a positive childbirth experience. Geneva, 2018. 30070803

[11] Shakibazadeh E, Namadian M, Bohren MA (2018). Respectful care during childbirth in health facilities globally: a qualitative evidence synthesis. BJOG.

[12] Maya ET, Adu-Bonsaffoh K, Dako-Gyeke P (2018). Women’s perspectives of mistreatment during childbirth at health facilities in Ghana: findings from a qualitative study. Reprod Health Matters.

[13] Bohren MA, Mehrtash H, Fawole B (2019). How women are treated during facility-based childbirth in four countries: a cross-sectional study with labour observations and community-based surveys. The Lancet.

[14] von Dadelszen P, Payne B, Li J (2011). Prediction of adverse maternal outcomes in pre-eclampsia: development and validation of the fullPIERS model. The Lancet.

[15] Payne BA, Hutcheon JA, Ansermino JM (2014). A risk prediction model for the assessment and triage of women with hypertensive disorders of pregnancy in low-resourced settings: the miniPIERS (Pre-eclampsia Integrated Estimate of RiSk) multi-country prospective cohort study. PLoS Med.

[16] Ce Drechsel K, Adu-Bonsaffoh K, Olde Loohuis K, et al. Maternal near-miss and mortality associated with hypertensive disorders of pregnancy remote from term: a multicenter observational study in Ghana. AJOG Global Reports. 3. 10.1016/j.xagr.2021.100045 PMC9564034 36275498

[17] Adu-Bonsaffoh K, Tamma E, Nwameme AU (2022). Health professionals’ perspectives on clinical challenges in managing hypertensive disorders of pregnancy and recommendations for improving care: a multi-center qualitative study. Front Glob Womens Health.

[18] Tamma E, Adu-Bonsaffoh K, Nwameme A (2023). Maternal hypertensive mother’s knowledge, attitudes and misconceptions on hypertension in pregnancy: a multi-center qualitative study in Ghana. PLOS Global Public Health.

[19] Cluver C, Novikova N, Koopmans CM (2017). Planned early delivery versus expectant management for hypertensive disorders from 34 weeks gestation to term. Cochrane Database Syst Rev.

[20] van Oostwaard M, van Eerden L, de Laat M (2017). Maternal and neonatal outcomes in women with severe early onset pre-eclampsia before 26 weeks of gestation, a case series. BJOG.

[21] Braun V, Clarke V (2006). Using thematic analysis in psychology. Qual Res Psychol.

[22] Carter N, Bryant-Lukosius D, DiCenso A (2014). The use of triangulation in qualitative research. Oncol Nurs Forum.

[23] Tong A, Sainsbury P, Craig J (2007). Consolidated criteria for reporting qualitative research (COREQ): a 32-item checklist for interviews and focus groups. Int J Qual Health Care.

[24] de Azevedo DV, de Araújo ACPF, Clara Costa ĹC (2011). An analysis of the meanings of pre-eclampsia for pregnant and postpartum women and health professionals in Rio Grande do Norte, Brazil. Midwifery.

[25] Carter W, Bick D, Mackintosh N (2017). A narrative synthesis of factors that affect women speaking up about early warning signs and symptoms of pre-eclampsia and responses of healthcare staff. BMC Pregnancy Childbirth.

[26] You WB, Wolf M, Bailey SC (2012). Factors associated with patient understanding of preeclampsia. Hypertens Pregnancy.

[27] Tsigas E. Preeclampsia: the patient perspective. In: NIH’s National Institute o f Child Health and Human Development (NICHD) workshop. Preeclampsia: A Pressing Problem. 2006.

[28] Sotunsa JO, Vidler M, Akeju DO (2016). Community health workers’ knowledge and practice in relation to pre-eclampsia in Ogun State, Nigeria: an essential bridge to maternal survival. Reprod Health.

[29] Wotherspoon AC, Young IS, McCance DR (2017). Exploring knowledge of pre-eclampsia and views on a potential screening test in women with type 1 diabetes. Midwifery.

[30] Leeners B, Neumaier-Wagner P, Kuse S (2007). Emotional stress and the risk to develop hypertensive diseases in pregnancy. Hypertens Pregnancy.

[31] Yu Y, Zhang S, Wang G (2013). The combined association of psychosocial stress and chronic hypertension with preeclampsia. Am J Obstet Gynecol.

[32] Kurki T, Hiilesmaa V, Raitasalo R (2000). Depression and anxiety in early pregnancy and risk for preeclampsia. Obstet Gynecol.

[33] Caplan M, Keenan-Devlin LS, Freedman A (2021). Lifetime psychosocial stress exposure associated with hypertensive disorders of pregnancy. Am J Perinatol.

[34] Danso KA, Opare-Addo HS (2010). Challenges associated with hypertensive disease during pregnancy in low-income countries. Int J Gynecol Obstet.

[35] Knight HE, Self A, Kennedy SH (2013). Why are women dying when they reach hospital on time? A systematic review of the ‘third delay’. PLoS ONE.

[36] Pacagnella RC, Cecatti JG, Parpinelli MA (2014). Delays in receiving obstetric care and poor maternal outcomes: results from a national multicentre cross-sectional study. BMC Pregnancy Childbirth.

[37] Witter S, Garshong B (2009). Something old or something new? Social health insurance in Ghana. BMC Int Health Hum Rights.

[38] Okong P, Byamugisha J, Mirembe F (2006). Audit of severe maternal morbidity in Uganda—implications for quality of obstetric care. Acta Obstet Gynecol Scand.

[39] Schutte JM, Schuitemaker NWE, Van Roosmalen J (2008). Substandard care in maternal mortality due to hypertensive disease in pregnancy in the Netherlands. BJOG.

[40] Bohren MA, Vogel JP, Hunter EC (2015). The mistreatment of women during childbirth in health facilities globally: a mixed-methods systematic review. PLoS Med.

[41] Rominski SD, Lori J, Nakua E (2017). When the baby remains there for a long time, it is going to die so you have to hit her small for the baby to come out: justification of disrespectful and abusive care during childbirth among midwifery students in Ghana. Health Policy Plan.

[42] Crissman HP, Engmann CE, Adanu RM (2013). Shifting norms: pregnant women’s perspectives on skilled birth attendance and facility-based delivery in rural Ghana. Afr J Reprod Health.

[43] Moyer CA, Adongo PB, Aborigo RA (2014). ‘They treat you like you are not a human being’: maltreatment during labour and delivery in rural northern Ghana. Midwifery.

[44] de Azevedo DV, de Araújo ACPF, Costa ÍCC (2011). An analysis of the meanings of pre-eclampsia for pregnant and postpartum women and health professionals in Rio Grande do Norte, Brazil. Midwifery.

[45] Værland IE, Vevatne K, Brinchmann BS (2016). An integrative review of mothers’ experiences of preeclampsia. JOGNN J Obstet Gynecol Neonatal Nurs.

[46] Leiferman J, Sinatra E, Huberty J (2014). Pregnant women’s perceptions of patient-provider communication for health behavior change during pregnancy. Open J Obstet Gynecol.

[47] Leeners B, Rath W, Kuse S (2006). Satisfaction with medical information in women with hypertensive disorders in pregnancy. J Psychosom Res.

[48] Barlow JH, Hainsworth J, Thornton S (2008). Women’s experiences of hospitalisation with hypertension during pregnancy: feeling a fraud. J Reprod Infant Psychol.

[49] Lewis R, Sibai B (1997). Recent advances in the management of preeclampsia. J Maternal-Fetal Med.

[50] Helewa M, Heaman M, Robinson M-A (1993). Community-based home-care program for the management of pre-eclampsia: an alternative. CMAJ Can Med Assoc J.

[51] Barton JR, Stanziano GJ, Sibai BM (1994). Monitored outpatient management of mild gestational hypertension remote from term. Am J Obstet Gynecol.

[52] Perry H, Sheehan E, Thilaganathan B (2018). Home blood-pressure monitoring in a hypertensive pregnant population. Ultrasound Obstet Gynecol.

